# Exploring homecare leaders’ risk perception and the link to resilience and adaptive capacity: a multiple case study

**DOI:** 10.1186/s12913-024-10808-4

**Published:** 2024-03-14

**Authors:** Ingvild Idsøe-Jakobsen, Heidi Dombestein, Siri Wiig

**Affiliations:** SHARE - Centre for Resilience in Healthcare, Faculty of Health Sciences, University of Stavanger, N-4036 Stavanger, Norway

**Keywords:** Adaptive capacity, High-quality care, Homecare, Leadership, Resilience, Risk perception, Sensemaking, Work-as-done, Work-as-imagined

## Abstract

**Background:**

Home-based healthcare is considered crucial for the sustainability of healthcare systems worldwide. In the homecare context, however, adverse events may occur due to error-prone medication management processes and prevalent healthcare-associated infections, falls, and pressure ulcers. When dealing with risks in any form, it is fundamental for leaders to build a shared situational awareness of what is going on and what is at stake to achieve a good outcome. The overall aim of this study was to gain empirical knowledge of leaders’ risk perception and adaptive capacity in homecare services.

**Methods:**

The study applied a multiple case study research design. We investigated risk perception, leadership, sensemaking, and decision-making in the homecare services context in three Norwegian municipalities. Twenty-three leaders were interviewed. The data material was analyzed using thematic analysis and interpreted in a resilience perspective of work-as-imagined versus work-as-done.

**Results:**

There is an increased demand on homecare services and workers’ struggle to meet society’s high expectations regarding homecare’s responsibilities. The leaders find themselves trying to maneuver in these pressing conditions in alignment with the perceived risks. The themes emerging from analyzed data were: ‘Risk and quality are conceptualized as integral to professional work’, ‘Perceiving and assessing risk imply discussing and consulting each other– no one can do it alone’ and ‘Leaders keep calm and look beyond the budget and quality measures by maneuvering within and around the system’. Different perspectives on patients’ well-being revealed that the leaders have a large responsibility for organizing the healthcare soundly and adequately for each home-dwelling patient. Although the leaders did not use the term risk, discussing concerns and consulting each other was a profound part of the homecare leaders’ sense of professionalism.

**Conclusions:**

The leaders’ construction of a risk picture is based on using multiple signals, such as measurable vital signs and patients’ verbal and nonverbal expressions of their experience of health status. The findings imply a need for more research on how national guidelines and quality measures can be implemented better in a resilience perspective, where adaptive capacity to better align work-as-imagined and work-as-done is crucial for high quality homecare service provision.

**Supplementary Information:**

The online version contains supplementary material available at 10.1186/s12913-024-10808-4.

## Background

The efficient deployment of resources in home-based healthcare is considered crucial for the sustainability of healthcare systems worldwide [1]. The share of patients expected to be treated at home is increasing in Norway and many other Western countries [2–5]. This is mainly due to policy initiatives to reduce the use of and the length of stays in hospitals and long-term residential care facilities [1, 6–8] and because people want to age at home as long as they are capable of [9]. The healthcare provided in the homes is becoming progressively more complex to attain supportive, rehabilitative, curative, and comforting purposes [6, 10].

The high volume of homecare patients and the complex system of service provision in these settings is also implying high standards in terms of collaboration and communication among healthcare professionals [11–13]. The leaders of homecare services play a key role in communication, trust-building, establishing a sound patient safety culture, promoting an innovation-oriented mentality, and in encouraging frontline healthcare workers to provide safe and high-quality care [14–19]. Allocating scarce resources to homecare services is a leader’s responsibility and often a challenge to complete in everyday practice with constant changes and pressures. However, knowledge of how homecare leaders’ work to align demand and capacity challenges is limited [20–22].

Further, caring for patients in their homes is multifaceted and conceptually different from hospital care [11]. Homecare services must handle an almost overwhelming amount of information daily [12, 13]. However, despite the increasing life expectancy worldwide and the substantial increase in the projected number of older adults using homecare, less attention has been paid to patient safety within the context of homecare services [6, 17, 23–26], especially compared to research on patient safety in hospital care [11, 27, 28].

In the homecare context, risk of adverse events exists due to for example error-prone medication management processes and prevalent healthcare-associated infections, falls, and pressure ulcers, making the issue an essential field for future research [10, 24, 29–31].

There is a significant body of research literature on how leaders affect patient safety culture among healthcare professionals and patient safety outcomes [16, 18, 32, 33]. In this study, we address how healthcare leaders make sense of the risk picture that develops in this part of the healthcare service. We do not define leaders or leadership in terms of a specific style, such as transformational, transactional, complex, or contingency leadership, just to mention some, as this is outside the scope of our study. However, we do acknowledge the important role of the leaders of complex organizations such as homecare.

### Safety I, safety II, and adaptive capacity

In healthcare, patient safety is often associated with quality [14], and the traditional approach to managing risk and safety in healthcare has previously focused on counting the number of errors to understand why things go wrong and establish barriers and procedures to prevent them. This approach refers to Safety I [34]. Predicting future adverse events is difficult, so recent thinking leads to the argument that the Safety I approach is insufficient in facilitating safer services. There has been a call for understanding and explaining how everyday situations usually are managed with successful outcomes, as an additional foundation for improving patients’ safety [35–38].

The Safety II approach, also referred to as resilient healthcare, acknowledges that healthcare professionals can adapt and provide high-quality services despite a lack of resources, disruptions, and stress [35]. Clinicians adjust to the conditions in which they work by assessing individual situations daily [11, 12, 26]. Resilience in healthcare thus provides a theoretical perspective for understanding complex adaptive systems [14, 39–42]. A system’s capacity to adapt has been described as a foundation for the development of resilience in the healthcare sector. Lyng et al. (2022) addresses this issue and state that “*adaptive capacity in healthcare constitutes adaptations based on reframing, aligning, coping and innovating, in response to external and internal demands from different organizational levels, in order to ensure quality of care.” (43, page 8)*. The backbone of adaptive capacity in safety research is thus adjusting to potential damage and coping with the consequences [43]. Accordingly, work-as-imagined (WAI) and work-as-done (WAD) are key concepts in resilience in healthcare literature [44]. The world we can perceive/imagine/and plan for (WAI) differs from the real world in which we work (WAD). This gap is often due to a combination of work conditions that do not favor a set of procedures, individual differences, and competing priorities in the workplace [45–47]. From a strict Safety I perspective, this gap will often be undesirable, as the system is designed to avoid deviations. From a Safety-II perspective, the gap between WAI and WAD is not necessarily negative but might be a sign of adaptive capacity and resilience within the healthcare system. This is how workarounds can be based on continuous risk assessments and result in deviations but still be aligned with patient-centeredness, high quality, and patient safety [47, 48]. However, workarounds are dependent on mutual trust among leaders, healthcare professionals, and team members [43]. Professional training in risk assessment, patient safety competencies, and quality leadership within healthcare organizations are often missing [49].

### Risk perception

To perform adaptively, risk detection is crucial. When dealing with any risk, it is fundamental that all relevant parties and stakeholders build a shared situational awareness of what is going on and what is at stake. To fully achieve this, stakeholders need to establish a shared awareness of what is considered a good outcome, as this might differ among them. Equally important is knowledge of how information about any risk factor and its consequences can be sought and assessed very differently by stakeholders in particular situations, contexts, and under various conditions. According to Weick et al. [50], sensemaking involves turning circumstances into a situation that is comprehended explicitly in words, which serves as a springboard into action [51]. It is therefore key to appreciate that risk makes sense to people not uniformly, but diversely, and is both culture and context dependent [50, 52, 53].

While the term risk might be mathematically calculated in terms of the risk factors, the probability, and the consequences, risk perception as a term is all about people’s conceptualization of risk and disparities in how different groups of people experience it. Risk perception refers thus to both an individual’s and the cultural understanding of the phenomenon of risk [54]. How risk is perceived is imperative when understanding which risk factors that get attention and are acted upon and which do not, for example when healthcare professionals provide services in someone’s home [52].

Risk perception may not be conscious but might be based on heuristics and unspoken presumptions [52, 55]. This feature raises the importance of openly discussing what we think is going on and what is at stake in a homecare context. Nonetheless, studies on how homecare service leaders make sense of patient safety risks are lacking. Further, knowledge of adaptive leadership in the homecare context and leaders’ conceptualization and awareness of patient safety risks from a resilience perspective is also limited [33, 56]. This means that the ways in which leaders conceptualize and make sense of risk information, continuously prioritize, and make decisions, involve their employees, and deal and cope with emerging risks are still under-explored.

Connecting the terms risk and safety is crucial for patient safety practice. There cannot be safety work without addressing risks. In our study we put emphasis on a shared situational awareness, resilience, adaptive capacity, and trust. The rationale for our study is hence that how healthcare services are adapted to patient needs in collaboration with staff and patients are pivotal to patient safety.

### Aim and research question

Although the research and knowledge of homecare services as a part of the health system are growing, considerable gaps still exist. The current article addresses the gap between leadership and adaptive capacity in homecare from the perspective of Norwegian homecare leaders.

The overall aim of this study was to gain empirical knowledge of risk perception and adaptive capacity in the complex adaptive system of homecare. More specifically we investigate of how the concept of risk is perceived and used to guide the leaders in homecare health services when aspiring to attain resilient and high-quality healthcare. The following research question guided our study:

How do leaders in homecare settings make sense of patient safety risks, and how do they adapt their leadership actions accordingly when aspiring for high-quality care?

By integrating how leaders perceive and act on risk, this study highlights new ways of using resilience and Safety II lenses to improve healthcare quality and safety. The study results are analyzed and discussed within WAI and WAD frameworks [44]. Understanding how risk, as a concept, is perceived is important when addressing resilience and adaptive capacity in healthcare. By applying the WAI versus WAD to risk perception, we can gain important new insight into how leaders make certain assumptions and sense of the world and how they adapt and act on it.

## Methods

### Design and study setting

The study applied a qualitative research design using a multiple case study approach [57–61]. Case study design is preferred when separating the context from the case is not possible and the research questions includes asking how and/or why something is. Including more than one case is always preferable if possible to gain richer data [59]. We investigated the phenomenon of risk perception, leadership, sensemaking, and decision-making in the homecare services context in three Norwegian municipalities, defined as single cases [59].

No universal definition of homecare services has been established despite its pivotal role [8]. As homecare can be both formal and informal social care and healthcare [8, 12], we defined homecare services in this paper as formal healthcare provided by the municipal healthcare system in the Norwegian setting.

### The context– the Norwegian healthcare system

The Norwegian healthcare system is partly decentralized and organized according to the division of state, county, and municipality [62]. There are, in total, eleven counties, and 356 municipalities - the latter varying greatly in size and demography. The Norwegian healthcare service can further roughly be divided into primary and specialized. Norwegian municipalities are, by national law, responsible for primary care services. This includes the general practitioner services, nursing homes and homecare services, and other services provided over a long period in the local environment. The specialized healthcare services are, in contrary, mainly run by the state through regional health authorities as owners of hospital trusts [63].

While the state is responsible for ensuring regulations and financial frameworks, supervision, and control, the municipalities are responsible for providing their residents with good and sound health and social services, regardless of age or diagnosis [64]. The Municipal Health Services Act imposes the municipality to ensure that people who stay in the municipality are offered the necessary health and care services [65]. The municipality can choose whether it will employ personnel to carry out the necessary tasks or enter into an agreement with private organizations or individuals to provide the statutory services. Further, the internal organization is decided by the municipality itself, and there are quite large variations among different municipalities in how the health and social services are organized [63].

Within the Norwegian context, homecare is primarily healthcare services in the home. The requirements for being granted home-based healthcare are dual. First, the patients must be dependent on help with activities of daily life (ADL) to cope at home, and/or they cannot get the healthcare elsewhere, besides the hospital. ADL tasks that are frequently aided are getting out of the bed in the morning, visiting the toilet, getting dressed and similar activities. Help with safe medication administration is moreover a typical need [3]. Practical aid, such as help with domestic work can also be provided by the municipality, but this is not referred to as “homecare” but rather referred to as help at home (e.g., cleaning), but not related to healthcare services.

### Recruitment

To obtain richness in data, we wanted to recruit more than one case municipality to our study. We considered three cases to be both sufficient to gain in depth insight into diverse setting and variety in locations, and manageable in terms of data collection and analysis of results across cases. In Norway, living conditions, socio-economic status and age distribution are relatively similar and equally spread across the municipalities. When recruiting municipalities to our study, we therefore had no inclusion or exclusion factors regarding such issues.

In the process to recruit three municipalities, seven were invited in total. The invitations were all sent to the municipal healthcare managers by email. If the municipal healthcare manager responded positively, the municipality was invited to a digital information meeting with the first author of the study. The municipal managers for health and representatives of all leader levels within the homecare departments attended these information meetings.

Besides given information about the aim and design of the planned study, the municipalities were asked to facilitate the interviews to fit their schedule. We asked for at least six interviews in each municipality. It was emphasized that all leader levels within the municipal homecare service should be represented in the study. The municipalities were ensured that they would not be asked about leader skills or person-sensitive information of any kind.

The four municipalities that declined the first invitation did so for diverse reasons, such as that they were in a demanding reorganization or could not set aside the necessary time for the study for other reasons. All the municipalities that responded positively to the first email and attended an information meeting accepted the invitation to participate in the study. All participation in interviews was voluntary and this was highlighted before all interviews.

### Participant characteristics

In terms of location, two municipalities were in the western part of Norway, and one was in the southern part of the country. The three recruited municipalities all have both city centers and rural areas, but the population size and the size in square kilometers differ, as illustrated in Table 1 below.

**Table 1:** The characteristics of the municipalities

	Municipality 1	Municipality 2	Municipality 3
Size	Less than 500 km^2^	500-1.000 km^2^	More than 1.000 km^2^
Population	More than 100.000	20.000–50.000	20.000–50.000

In Norway there has been a reorganization reform for better management of the municipalities. It has been the Norwegian government’s view that larger municipalities are better in terms of administration, finances, and fairer distribution of public services. The three participating case municipalities had all individually been part of a merger process, where each of them had been merged with surrounding municipalities.

The municipalities organized the homecare in multiple units and departments located apart from each other, functioning as base stations for the healthcare professionals and the middle leaders. One municipality has coordinators, department leaders, and unit leaders; one has department leaders and unit leaders; and one has assistant department leaders and department leaders but no unit leaders. All the municipalities have a municipal manager for the health sector. All leader levels were represented in the study. The average department leader interviewed had about 20–40 employees in his/her department at the time of the study. Almost every leader interviewed in the study was female, and all except three were registered nurses.

### Data collection

Data was collected through individual interviews. The first author conducted interviews from May to November 2022. The interviews were conducted at local sites of the homecare units, except for one interview, which was conducted digitally via Microsoft Teams. The interviews lasted about an hour each and were recorded and transcribed verbatim. The interviews were semi-structured, all guided by the same interview guide. The theoretical framework of risk perception, social amplification of risk, and work-as-imagined and work-as-done [44, 66, 67] inspired the interview guide containing the following sections:

- General information about the work tasks, education, and experience.
- Open questions about how they think of risk and patient safety as concepts and how they use it in leadership.
- Open questions about how the municipalities organize, plan, and adapt healthcare services.
- Open questions about how they see quality measures, legislation, and guidelines implemented by the government.
- Open questions about how they find the expectations from society and how they cope with these expectations.
- Open questions about quality and high-quality care.
- Open questions about the homecare strengths and limitations.

The leaders’ answers guided further follow-up questions. In particular, details about the processes and personal thoughts about adjusting the planned work were followed up when relevant and appropriate.

In sum 23 individual semi-structured interviews were conducted with leaders in the three municipalities. Table 2 shows the number of interviews in each municipality and when they were conducted:

**Table 2:** Time and number of interviews

	Time of interviews	Number of interviews
Municipality 1	May 2022	8
Municipality 2	September and October 2022	8
Municipality 3	September and November 2022	7
In total		**23**

Because the homecare services were organized differently in the three case municipalities, the informants had different titles and responsibilities as leaders. However, all leader levels within the municipality were represented in the sample. The three leader levels above the team coordinators and assistant Heads of Department all had personnel responsibility. Table 3 shows the leader levels within each municipality, starting at the top leader level in the study:

**Table 3:** Levels of leaders interviewed

	Municipality 1	Municipality 2	Municipality 3
Municipal manager for health	x	x	x
Unit manager	x	x	
Head of the department	x	x	x
Assistant Head of Department			x
Team coordinator	x		

### Data analysis

Our interview guide was inspired by theoretical contributions, but our analysis was inductive, and data driven. Once transcribed and read, the interviews were uploaded and coded inductively in Nvivo. The data analysis was conducted according to Braun and Clarke’s six-phase thematic analysis [68]. The first phase of thematic analysis involved familiarizing oneself with the data [68], including conducting, transcribing, and reading and re-reading the interviews. In the second phase, interesting features of the data were coded systematically by searching for initial codes. In the third phase, these codes were then analyzed for themes. The initial codes were kept, renamed, discarded, or merged multiple times in the fourth phase. The process was repeated with the themes and subthemes that had emerged from the initial codes in the fifth phase. The final sixth phase involved naming the themes, and sub-themes were set and re-set when writing the report. This represents the last analytical step in the content analysis [68].

A multiple case study research design entails both within-case-analysis and across-case-analysis [59]. This was also done in this study. The data analysis was conducted as a within-case-analysis in phases 1–3. Cross-case-analysis was conducted in phases 4–6. However, the data was not merged, but kept easily identifiable throughout the entire analysis.

The first author led the analysis and conferred with the other authors throughout the six phases of the content analysis. The author team agreed on the final three themes through discussions, as described in the results.

## Results

The three overall themes emerging from the analyses included several distinct sub-themes that shed light on leaders’ experiences of risk as a concept and academic term and their use of it as a guide to leadership. Table 4 summarizes the themes, subthemes, and codes from the analyses:

**Table 4:** The themes, subthemes, and codes

	Themes	Sub-themes	Codes
1	Risk and quality are conceptualized as integral to professional work	Making sense of necessary healthcare despite a lack of definition	Necessary healthcare as the core task
			The clinical gaze
		Making room for the staff’s professional assessments	Sharing information and discussing
			Person-centredness
2	Perceiving and assessing risk imply discussing and consulting each other– no one can do it alone.	Work-as-imagined– Planning the work but preparing for deviations	Assignment and work list as logistics and planning tool
		Work-as-done– Continuous adaptation in practice	Flexibility in the service and the work list
			Information flow, individual and joint sensemaking, and decisions
		Weighing risk and patient-centredness	The care receiver’s free will and integrity
			High-quality care and working environment
3	Leaders keep calm and look beyond the budget and quality measures by maneuvering within and around the system.	Making sense of the protocols	The financial model
			The reporting system
			Regulations, legislations, and national guidelines
		Defining leadership	Taking care of the staff
			Dealing with the context

In the following section, we illustrate how leaders used their risk perception to adapt their leadership actions to attain high-quality care. We show how the homecare services struggle to meet the high expectations from society regarding homecare’s responsibilities, and the increased demands on the homecare workforce, which leaves these services to work within a rather demanding context. The leaders find themselves trying to navigate these pressing conditions to allocate scarce resources in the homecare in light of the risks as they perceive them.

### Theme 1 - Risk and quality are conceptualized as integral to professional work

Although most of the leaders themselves did not use the word “risk” under the interviews, we still found that they were talking about the issue, but in other words. In theme 1, we present how the leaders argued for how they made sense of both risk and quality within homecare services.

#### Making sense of necessary healthcare despite a lack of definition

The results showed that the heads of department and coordinators are unaccustomed with using the risk concept. They addressed risk differently in practice, rarely referring to the issue by phrasing the term risk. Instead, they addressed the topic when talking about the clinical gaze and monitoring changes in the health status of the care receivers to make sure early symptoms and signs of illness and deteriorating health status were properly captured, and relevant measures and interventions were introduced.

Accordingly, the leaders naturally utilized terms synonymous with the risk that referred to observation, making a considered decision, and assessing and being ready and able to act based on intentionally sought information. In practice, leaders and healthcare professionals linked risk information and risk perception to professionalism without using the term risk:

It is like a holistic assessment which is part of being a nurse or healthcare worker; you have the eye for such things when you enter the home, the subject, the expertise and the experience, and a sense of that gut feeling. […] We should never trivialize things we do in the homes, […] if you have a good gaze, you can prevent quite a lot (leader of Municipality 1).

In addition, instead of using the word risk, the informants spontaneously used the terms ‘being in danger of’ or something being ‘professionally liable’. These phrases were often related to something specific, like being in danger of falling, developing urinary tract infections, or being in danger of malnutrition.

The leaders also conceptualized high-quality care as seeing and caring for the person as an individual:

I would say that we provide good quality services here. We do. We can serve most people in terms of how the patient and relatives prefer it, if these wishes are reasonable, of course (leader of Municipality 2)

The leaders emphasized that one size does not fit all when it comes to meeting the patient’s needs and delivering high-quality care. The leader’s task is to create and retain a working environment that promotes sharing information and discussions among the healthcare professionals about the patient’s health status. The leaders said the concerns and good ideas put forward by their employees are of key importance.

The leaders drew on their understanding of risk and patient safety as professionals and believed that their employees were perfectly capable of delivering sound healthcare due to their training. The results showed that the leaders, in general, did not have the impression that the guidelines, legislation, or national statistic programs guide them when aspiring for high-quality care. The national professional guidelines for malnutrition are one concern. The guidelines require homecare caregivers to assess nutrition in all cases, which the leaders reported as time-consuming and invading the patients’ lives. The inflexibility of the guidelines was, in the leaders’ opinion, a devaluation of the professional caregivers’ competence. However, the leaders supported that the national authorities should make regulations and guidelines and found the topics important but expressed that the authorities failed to consider the overall effects of the requirements. Sometimes the total regulatory burden made the homecare leaders think that complying with the legislation come at the expense of the care given in the homes.

The results also demonstrated a lack of a shared definition and understanding of ‘necessary healthcare’. The leaders in our study reported increased expectations from the public, including the impression that homecare is responsible for the well-being and everyday lives of home-dwelling persons. This frustrated the leaders as the expectations could not be met in homecare practice:

So, what can you expect from the homecare services? What can you expect the municipality to offer? It’s like, we must provide the ‘necessary healthcare’, and that definition is very broad. What does that even mean? Some people might think that it means that we will shop for them, we will accompany them to the doctor, and we will buy them clothes and make sure that their house is spotlessly clean, and that the windows are polished, and, you know, they want it all (leader of Municipality 1).

The leaders also argued that not having a shared definition of ‘necessary healthcare’ seemed to increase the expectations from the public over time.

In addition, the leaders argued the public healthcare system is generally expected to be responsible for the well-being and everyday lives of home-dwelling individuals, including the social well-being of the patients and their daily activities, like visiting the hairdresser, getting the mail, paying bills, setting up the TV remote, throwing the trash, and doing the laundry. The leaders shared two perspectives. They acknowledged that housework and a good social life are important parts of good quality of life, and they would like to contribute to their patients’ lives in line with a person-centered approach to healthcare, but they pointed out the lack of time, resources, or healthcare professionals to conduct this kind of work.

#### Making room for professional assessments

The leaders usually talked about risk and risk assessments without using these terms. Instead, they talked enthusiastically about the staff’s clinical gaze. Capturing changes in the patients’ health condition was by some leaders presented as what it was all about.

Because it’s kind of, it’s our job, it’s what we do, right, […] observing for change, that’s what nursing is, in a way, that’s what both nurses and nurse assistants do, it’s the observations that lead to an action or no action, but it should be, in a way, reflected around, it should be a considered decision, it’s not something, you can’t just overlook things, you have to have made an assessment. What I’ve seen today, is it going to lead to something or is it not going to lead to something? Should I do something about it, or should I not do anything about it, is there something that needs to be documented, or is it completely unnecessary, is this within the normal range, or is there a change, and those assessments. Yes, that is what they do all the time and they do it more or less automatically, so that it’s not necessarily conscious, like, now I sit down and take the assessment, that’s our job, that’s why we’re at work (leader of Municipality 1).

According to the leaders, the healthcare professionals feel morally, ethically, professionally, and personally obliged to care for the patients in a person-centered way. However, in practice, they found it difficult owing to continuous trade-offs due to scarce resources. This left the healthcare professionals feeling guilty for refusing or not taking the time to conduct domestic work, such as cleaning the floor, emptying the dishwasher, and doing laundry in their patients’ homes.

I often tell my employees, if you are worried about patients, worried about relatives, then I say, we will help with that, we will help them with what they need, but we cannot carry them in our arms, it is their lives. So, from time to time we must spend time, as leaders, talking about the limits of the homecare and what we can expect from the relatives (leader of Municipality 3).

The leaders’ role was to support their employees in this struggle and, in some cases, contribute to the discussion among the healthcare professionals and walk the extra mile for the particularly vulnerable patients lacking family or a social network to support them. The leaders emphasized that it is not the job of the public health system to do the dishes, pay bills, and do grocery shopping, but they can assist the patients in getting help elsewhere. However, as the leaders said, when there is no food in the fridge, the floors are dirty and sticky, and there is a nasty smell leaking from the trash bin, it is necessary to help.

Homecare should be restricted to vital healthcare, although assessing when and where this extra care, which requires continuous risk assessments in practice, is vital to care and was found integral to their professional judgment.

### Theme 2 - Perceiving and assessing risk involves discussing and consulting each other– no one can do it alone

Planning the work thoroughly was indeed an important part of providing high-quality care. However, as the leaders emphasized during the interviews, a plan might not be adequate, feasible or best practice for a long time. In theme 2 we present the leaders views on how adapting care to the reality affects care quality.

#### Work-as-imagined– planning the work

When planning the work, homecare services receive medical and additional information about the patients from several sources, like hospitals, the patient’s doctors, families, and nursing homes. As a starting point, the municipal resource allocation office gives the homecare services a letter including the decision on healthcare services to be delivered to the patient by the homecare.

Meanwhile, when the homecare services visit patients at home, it is often the first time a person from the health services maps patients in their natural surroundings. The results showed that the leaders believed they often received inadequate or no longer up-to-date introductory information and written decisions about the patient’s actual needs. Thus, the homecare services made their assessments before the service started based on the order from the resource allocation office as a starting point:

And when they [resource allocation office] have concluded, you know, what is the right service level, then they say, ok, this person needs homecare for very specific tasks, like medication administration, for example, or help with getting dressed in the morning and ready for bed in the evening, and then they write a decision, and then we get that person home. As a rule of thumb, the decision does not match the actual needs, though […] very rarely. Some decisions are very specific when they are discharged from the hospital, for example, while in other cases, we must just get started and then adapt a great deal (leader of Municipality 1).

After assessing patients’ healthcare needs, the leaders or coordinators planned the workday accordingly. The planning and the logistic aspects related to the kind of health service that had to be delivered at specific times, driving or walking distance to the patient, number of visits per shift, day or week, the competence needed of the health personnel, time per visit estimated and more practical elements like meetings and reports, lunch breaks, and length of the healthcare professionals’ workday were integrated into work lists. Planning and allocating the resources were mainly the coordinators’ or assistant heads of departments’ jobs who found estimating time per task according to the job list very difficult. Patients who depended on receiving help to get out of bed were prioritized before getting dressed. This exemplifies the issue of not being able to help all the patients simultaneously but establishing some queuing order.

Regarding how the homecare organized the work lists in terms of ambulant teams and dedicated work lists to nurses, nurse assistants, or unskilled assistants only, the results varied. Allocating the healthcare professionals according to competence or the number of patient visits were two considerations when distributing the work lists. In either principle, the leaders had to balance the shortage of nurses, risking missing out on important information due to competence or habits and having too many professionals visiting each patient.

#### Work-as-done implies continuous adaptation in practice

Coherent with planning the work, the homecare must adapt accordingly to the continuous changes in the health status of the patients and their need for assistance to live safely in their homes. The leaders reported that changing the work lists and the organization on very short notice happened every day.

The changes might be due to a patient’s unexpected decline in health status, implying more time spent on a specific assignment and falling short of their work duties. Other causes for changes in the work list could be new patients coming in on short notice or administrative tasks that had to be done, like writing reports; changing care plans; consulting with the patient’s doctor, families, and hospital; or aligning changes to the medication list.

Every day there are tasks related to communication with the doctor, hospital, and the resource allocation office due to changed patient needs. This kind of communication often means that the notices have to be followed up by us. If there are medication changes, that means something new have to be entered into our system, and perhaps we need to change the dose or order a new multidose. […]. So, a single notice in the system can imply four hours of work. There are a lot of follow-ups (leader of Municipality 2).

If the health status among the patients changed profoundly, the results showed that homecare professionals did what they found necessary and did not wait for permission from the resource allocation office. The priority was delivering sound healthcare and administering the paperwork afterwards. The priority was the patients’ needs here and now, not the estimated time in the work list. To adapt and adjust to healthcare individually, the leaders spoke of the homecare services as an organization where the healthcare professionals talk about their patients, share concerns, discuss measures and, in general, make collective sense of the overall information available. The leaders emphasized that every employee must be capable of making their own decisions when on an assignment, and when hiring, the leaders actively seek healthcare professionals that seem independent and robust. However, the leaders also added the important aspect of discussing and sharing information, highlighting that the individual healthcare professionals assess the health status of the patient differently and that the best practice is achieved when cooperating and making shared decisions.

In practice, all kinds of report meetings and even lunch breaks were used as arenas for discussing today’s experiences, impressions, and concerns. Calling each other to consult with a case, and occasionally also the leader, was an everyday practice. The results showed that the leaders did not think of these meeting places and sharing information as something they have initiated or implemented but spoke of them in terms of professionalism and commitment, which they welcomed and gladly facilitated. The result also showed that the IT systems were not well suited for the nature of homecare work. Written reports did not provide sufficient information and left the healthcare professionals in more demanding working conditions. When combining this with an ever-growing amount of administrative work, high expectations, and a financing model that sometimes was more of a barrier straining the system, this needed to be compensated by something. The leaders argued that the healthcare professionals were the compensating factor adapting to demands:

Why does it work? Why on earth? It’s like, why, what? None of us have the overview, so why is it working? […] Personal aptitude, perhaps. And it’s obvious, this work is so unpredictable, and things change so quickly, and people constantly must put aside what they had planned, you constantly must add on something extra, a phone rings, you get told to do this and that. You never get to finish anything you start in a day’s work. […] I think there is a sense of responsibility for yourself and others as well, and so it is like, it depends on the person, cause some are very good at it, some may not be so good at it, they just come to work and do their job for the day, and then they go home, but then it doesn’t add up, it doesn’t work. It depends entirely on people being flexible (leader of Municipality 1).

The leaders also elaborated on the assessment of sound professional practice relating to their patients staying at home. Patients’ ability to stay alone often differ from how they feel about being left alone. The leaders experienced that many patients felt unsafe and feared being alone, but the professional judgment indicated an acceptable risk level. The results moreover showed that despite not being responsible for allocating services, homecare services did assess risk all the time when weighing risks as health status, housing, family situation, social network, anxiety, risk of falling, risk of walking outside, not finding the way back home, risk of fires, and other factors. The latter argument pinpointed the dual role of the homecare. Although the resource allocation office grants or denies a short-term or long-term stay at a nursing home or care home, it is the homecare services that continuously conduct the everyday assessments of soundness.

#### Weighing risk and patient-centredness

The results showed that aligning the assessment of general risks and individual adaptation could result in ethical dilemmas. The leaders expressed that the homecare services do offer sound healthcare and that they find solutions if the situation changes drastically in an unsafe direction. The struggle was that the feeling of being unsafe is something the homecare leaders often cannot alter, and the leaders felt that the professional health system fell short. Homecare can relieve the situation to some extent by introducing digital welfare technology in the homes, but the results showed varying use of such technology. Alarms, geofences, and medication dispensers do not prevent all adverse events, but the alarms, in particular, help detect those events.

Another challenge is the group of patients that did not want to receive the healthcare they needed. The frustration and dilemma here were not that the homecare could not deliver sound healthcare, but rather that care quality also includes listening to the patient and not overstepping their boundaries, intimacy, and integrity, although the healthcare professionals might want to do more. Patient-centredness is also related to not over-engaging in the patients’ lives and health but being responsive to the expressed patient needs.

There was a dual understanding of risk related to a tight joining of what is safe for both the caregiver and the care receiver. The term risk typically implies the risk for the caregiver, distinguishing it from professional care and patient safety. However, the leaders reported that the caregiver’s and the care receiver’s risk was equally weighted in practice. In situations where high-quality care was impossible because of the demanding working environment caused by the care receiver, both the leaders and the healthcare professionals struggled.

Dealing with assessing the risk of violent attacks, feelings of insecurity, and not getting sufficient information to give sound care gave the leaders an experience of not fulfilling their obligations as leaders. They wanted to make proper assessments but did not find themselves in a position to implement the right measures. From this perspective, the risk was caused both by the care receiver but also the system, the lack of proper measurements, and the other health services, in particular the psychiatric care, leading to an undefinable experience of threat among the homecare leaders on behalf of their employees.

### Theme 3 - leaders keep calm and look beyond the budget and quality measures by maneuvering within and around the system

A consistent theme during the interviews was that being a leader within the homecare setting meant translating demands and protocols to real life setting. In theme 3 we address the main issues emphasized by the leaders.

#### Making sense of the protocols

Regarding reporting systems and documenting events, the leaders differed heavily in expressed interests and level of implementation in their units. While some of the leaders found the reporting system to be stressful, negatively oriented, uncomfortable, and with limited expectations of results, a handful of them talked quite passionately about documenting and handling non-compliance, deviations, and adverse events, linking it to high-quality care and aspiring for safety and reduced risks. These leaders explained that they had taken the initiative to improve the reporting culture.

The homecare services’ and the municipality’s internal control, including written routines and procedures, checklists and internal guidelines, and deviation systems to report and process adverse events, affected systematic work with patient safety and risk management. Leaders explained that the guidelines and checklists, though time-consuming, were still beneficial to prevent adverse events. An example was checklists with potentially major effects on the quality level in the transitions from home, hospital, nursing home, short-term ward, and vice versa. Some of the homecare leaders talked about such transitions as situations where essential information about the patient, who was not handed over in time or incorrectly, had the potential to cause adverse events. Getting it all right the first time was therefore preferred.

#### Defining leadership

The leaders talked about the financial model and budget as an issue they must deal with, but they also needed to look beyond to be a good leader for both their employees from a workload perspective and to enable the homecare services to be a flexible organization for the patients. The financial model in the three participating municipalities differed, although they all struggled with allocating resources due to the challenges in predicting the number of patients, the level of healthcare needed, and sick leave. In Municipality 1, in particular, the leaders expressed frustration about the financing model, with the resource allocation office linking geography and decisions. This model implied that homecare services always needed to enact a dual perspective when delivering healthcare. First, healthcare must be based on real needs, thus changing with the shifting health status of the patients. Second, the homecare must also send the resource allocation office change notices. This produces administrative work that the leaders find challenging as it reduces time spent with the patients.

To enable the flexible, resilient, and adapting organization, which was necessary to cope with swift changes, the leaders needed to grasp a wider horizon to get the full picture of their job as both a leader for their employees and to meet the demands of healthcare in the homecare context. The leaders could not turn their backs, financially speaking, on the problem, but they expressed a sense of coping and dealing with the situation by keeping calm and arguing why it was necessary to exceed the budget. Still, the leaders, in general, expressed frustration about what they interpreted as an unsolvable problem– budgeting correctly and not exceeding but still delivering healthcare that is ever-changing and complex:

We are left alone. We feel, like, with all the pressure from the regulations, from the Norwegian Parliament, from the health government, all the laws and regulations, it’s like, […], and on top of that, we get words from our management that the finances are so tight, we can’t afford anything extra, the economy is so stretched, so you have to try and manage as well as possible, but we can’t calculate how many will get sick and how many will die, can we? (Leader of Municipality 3).

## Discussion

The overall aim of this study was to gain empirical knowledge of risk perception and adaptive capacity in the complex adaptive system of homecare with an emphasis on information flow, sensemaking, and joint decision by the leaders. In the leader’s opinion, the full picture of a patient’s health status can be constructed based on different perspectives, and there is no such thing as an objective way of assessing the patients’ well-being and status.

The construction of a risk picture incorporates all the signals, both measurable vital signs and patients’ verbal and nonverbal expressions of their experience of health status. This means that the leaders recognize that people might perceive those early signs and symptoms of poorer health conditions differently. Some leaders spoke of this sensation and feeling as something that needed to be talked about, shared, and discussed to be capable of transitioning into something to act on. The homecare services must therefore position themselves to be capable of handling those early signs as a unit and not as individuals.

Due to this perception of risk, both a personal trait but also due to an aspect of education, experience and correlated with interests and time to perceive, joint sensemaking is a complex process. Work as imagined in our study, quite often turns out to be different to the work as done in practice– similarly to what as shown in the literature regarding WAI and WAD [69]. In line with previous research on how leaders contribute to adaptive capacity in hospital teams [39, 43, 70] and in nursing homes, we found that homecare leaders showed a high degree of flexibility, as they constantly worked for adaptive capacity, aspiring to attain high-quality care. Examples from practice related to rescheduled work lists to better suit the patient’s needs. Also emphasizing person-centeredness and listening to patients’ needs were highlighted. Furthermore, allowing the staff to go the extra mile for some patients also related to adaptive capacity.

The results revealed two positions of risk perception - risk as something that can be measured objectively (like vital signs) and risk as something more than what meets the eye (a subjective feeling something is wrong). In line with risk theory, these two positions have distinct characteristics and diversities in terms of what the risk is and how it can be perceived [52, 54, 71, 72]. Perceiving signals about the patient as a personal chemistry or personal trait with the healthcare professionals, nurses, and nurse assistants are similar in a figurative sense. The risk here refers to something subjective and more than vital signs and measurable symptoms, like an overall sensation, and quality care is about knowing the patients and being able to notice when the health status changes even subtly. Risk perception is here more like a feeling to catch. Assessing vital signs and somatic symptoms, as a matter of education and training, lingers on a positivist sense of science. This shows that the leaders in their everyday work balance different and sometimes opposing risk perceptions.

Emphasizing that the whole system must allocate services and later organize the healthcare soundly and adequately for each home-dwelling patient, the leaders take responsibility for empowering the individual nurse and nurse assistant, to acquire skills necessary to improve service quality, communication, and cooperation. Service quality, communication, and cooperation within the homecare setting are addressed also in previous research [12, 23, 26]. Further, we found that the leaders take responsibility for the whole system by always considering planned work as a starting point that should and must be changed if necessary. Our study demonstrated the key role risk perception when leaders adapt to align capacity and demands challenges. Both verbal and nonverbal signals (soft signals) from patients, families, and measurable signals of deterioration were highlighted. The same was complex physical environment in the home. The leaders continuously adapted service provision within the existing leeway or even made workarounds (e.g., beyond the limits of resource allocation office), to support the healthcare professionals and the service provision to the patients. This also implies, in line with social amplification of risk literature [66, 67], that risk elements for some patients were amplified (e.g., deteriorating patients without relatives) while other were attenuated and were considered to wait to be handled (e.g., shopping for patients with family nearby).

The homecare service is not just a performer of healthcare in an ordering-performing model. Since the leaders considered themselves and their employees as active participants in allocating healthcare, they assess risks and adapt the care accordingly without waiting for permission from the resource allocation office. The leaders’ job is to facilitate information flow and joint decision-making within the organization. This study participants addressed this issue during the interviews without using the term risk; instead, they spoke of the process as to be fond of the patients, wanting them to be safe and of their responsibility for delivering the best healthcare possible.

In our theoretical perspective, this is performing adaptively. The descriptions provided by the leaders aligned with how we define adaptive capacity [43], as they highlight making changes are necessary to provide high-quality care. Implying that the homecare leaders supported the staff to work in a resilient manner. Ensuring a work environment, where mutual trust and autonomy among leaders and staff was emphasized. When allowing and cheering changes in the work process (such as supporting staff spending more time with sick patients or taking out the trash for those who are not capable doing so themselves), the leaders manifested the basic idea for the WAI/WAD framework– that WAI does not always match WAD and adaptations are needed in practice to provide service quality. In this manner, what explicit terms related to risk used or not are interesting and need further study. We found that the leaders did not necessarily articulate concepts in a risk science vocabulary by using the terms “risk” or “risk factors”, but they showed a high awareness of risk, nonetheless through adaptations to patient’ needs, home situation, and family needs. Further studies should investigate this in other types of municipalities in a national and international setting to gain even deeper understanding of risk perception and adaptations in practice.

### Implications of the findings

Balancing the demands and resources in healthcare systems is a demanding task [20]. Multiple forces, variables, and influences must be factored into any change process [73] and implementation processes to promote a learning and resilient health system [31, 74, 75].

Our results showed that risk perception affects how the leaders not only organize the work when planning the work lists, routing of visits, and allocation of resources but also make sense of and prioritize national regulations, legislation, and guidelines. The leaders who do not find these quality measures aligned with their own risk reception might find it difficult to fully implement the measures and could experience a top-down system. Somehow, they find themselves talking another language than the authorities, which is also found in other literature on risk regulation [76, 77].

The leaders and the healthcare professionals show a capacity to adapt to the patients’ needs and the context, considering patient-centeredness, the financial model, and available resources. The healthcare system can profit from acknowledging the leaders’ competence and the healthcare professionals’ ability to incorporate risk assessments in their work, (work-as-done). The findings tell us that the leaders want to put the risks into words, which is also demanding and difficult because risk as a term appeared to be foreign, unfamiliar, and somewhat frightening, associated with external quality measures and legislation. By highlighting the value of their competence, both practice and research can learn from their high capacity to adapt. By doing so, risks in homecare services can be further explored.

The informants from the three municipalities did not differ greatly in how they perceived and talked about risk as a phenomenon, and one may question how the different context mattered. However, the results from the three municipalities differed in how informants acted on specific risks, such as (mal)nutrition, psychiatric care versus holistic person-centered care, use of welfare technology, and medication errors. This diversity in attention is also in line with the theoretical framework of social amplification of risk [66, 67, 78, 79]. Exploring and further measuring these issues is recommended as we believe it could add valuable knowledge on how specific risks are handled and acted upon in the homecare context.

In sum, the findings imply a need for more research on how national guidelines and quality measures can be implemented in a better way from change management and resilience perspectives, where adaptive capacity and the theoretical framework of work-as-imagined versus work-as-done are possible quality promoters and not barriers.

### Strengths and limitations

This study has strengths and limitations that need to be considered. First, the first author conducted all the interviews. This can be both a strength and a limitation. As a strength well worth emphasizing, the first author got in-depth knowledge about the local homecare departments. This knowledge made the interview situation easier for both parties, as not all background information had to be repeated in each interview. More time could hence be used on following up identified topics and issues. Following-up-questions are important when conducting semi-structured interviews [58], so this in-depth knowledge gained was pivotal. Although the interviews were conducted by one researcher within the researcher team, the interview guide was made as a discussion process within the researcher group. In terms of both reflexivity [80] and trustworthiness in general [60], we repeatedly discussed how to gain richness in data when the persons interviewed did not use the term “risk”. However, we find that the composition of the researcher team was a strength. The first author is a physical therapist,is trained in organizational theory, change management, risk management and auditing. The second author is a registered nurse well experience with the homecare setting and has a PhD in health science, and the last author is a professor of safety and quality in healthcare, well experienced in risk research and risk management. As the first author also led the initial three analysis phases in Nvivo, valuable information might have been overlooked and not followed up. However, we ensured trustworthiness by giving all the researchers a copy of the transcribed interviews and repeatedly discussing the results during the analysis in joint meetings and frequent communication by e-mail. As revising sub-themes and themes is a part of the six-phase analysis process [68] including the within and across case analysis, the researcher team collaborated closer in the last three phases of the analysis during the process of writing this paper.

Second, the results are based on detailed interview data from a sample of leaders from three different municipalities. A larger sample of municipalities and even larger sample of leaders could potentially add additional relevant aspect [59]. To ensure transferability of our results, we detailed the context and settings to allow other researchers to determine whether the results are relevant and transferable to other healthcare contexts, countries, and studies. However, further studies of municipalities with an even larger difference in geographical setting and location, and size is also recommended.

Third, our data collection lasted from May to November 2022. If we conducted a longitudinal study over several years, we could potentially see development or changes in ways risk is conceptualized and enacted upon. However, our study gave a clear and rich picture of the ways leaders understand and act based on risk information.

Fourth and last, the ethical considerations made throughout the study is worth elaborating on. To the best of our knowledge, all persons interviewed did so voluntarily. After the initial information meeting during the recruitment process, and prior to the interviews, we provided the municipalities with written information about the aim of the study. We here emphasized that participating was voluntary. We started all interviews with providing the informants with information about the study’s aim, that it was voluntary to participate, and let them know what we would and would not ask them about. We did not proceed with the actual interviews before we obtained informed written consent from the participants, both in terms of participating but also consenting to audio recording the interviews.

## Conclusions

In this study, we explored how homecare leaders make sense of patient safety risks and how they adapt their leadership actions accordingly when aspiring for high-quality care.

We found that leaders construct of a risk picture by incorporating a mixture of the signals, both measurable vital signs, family concerns, and patients’ verbal and nonverbal expressions of their experience of health status. Balancing these different points of views regarding the patients’ well-being indicates that the leaders do have a large responsibility for organizing the healthcare soundly and adequately for each home-dwelling patient. Risk information is complex and non-linear, and although the leaders did not use the terms risk or risk perception, discussing concerns and consulting each other about risk related factors was a profound part of the homecare sense of professionalism and leadership.

Our study showed that leaders and healthcare professionals were active participants, continuously assessing risks and adapting the care correspondingly without waiting for permission from the resource allocation office. This kind of adaptive capacity in everyday practice was crucial for homecare quality and safety. Dealing with risk was a large part of enacting homecare leadership. Facilitating information flow and joint decision-making within the organization, leaning heavily on work as planned, significantly differed from work as done. Through anticipating and monitoring risks, active involvement, trusting the competence, and adapting to patients’ needs, the leaders contribute to making the system adaptive and delivering high-quality care. However, more research is needed on how professionals continuously account for patients’ situations and risks, demonstrating adaptive capacity in the homecare setting.

### Electronic supplementary material

Below is the link to the electronic supplementary material.

Interview guide - translated version (from Norwegian to English)

## Data Availability

Original de-identified data can be requested from the corresponding author upon reasonable request. No database was used in the study.

## Abbreviations

WAI: work–as–imagined
WAD: work–as–done
