# Supplementary material for: Exploring homecare leaders’ risk perception and the link to resilience and adaptive capacity: a multiple case study
Source: BMC Health Serv Res. 2024 Mar 14;24:340. doi: 10.1186/s12913-024-10808-4 (PMC10941597; doi:10.1186/s12913-024-10808-4)
Supplement: Supplementary file 1 — Interview guide - translated version (from Norwegian to English) [file 12913_2024_10808_MOESM1_ESM.docx]

# Appendix 1 - Interview guide: Semi structured individual interviews

## 1. General background information

a. What is your current leader position in home care?

b. How long have you held the position?

c. What is your area of responsibility?

d. What is your education?

## 2. Risk perception and sensemaking

a. What does the term risk mean to you?

b. What does the term quality mean to you?

c. What does the term patient safety mean to you?

d. Within the homecare setting, do you work risk-based?

- If yes, what does the term mean to you?
- If yes, how?
- If yes, what are the pros and cons of working risk-based?

e. What kind of risks of patient harms / adverse events are known for you within the homecare setting?

f. What are the consequences if risks are overseen?

g. How do you identify and follow up risks in terms of quality care and patient safety?

h. How do you identify and follow up risk information regarding the individual patient?

i. What kind of systems and tools do you use (e.g., screening programs, guidelines, checklists, board, non-compliance, inquiry et cetera)?

## 3. Adaptive capacity and leadership

a. How are you involved in decisions concerning visit frequency and alterations of care plans?

b. What kind of information from the healthcare professionals do you need to make decisions and prioritize – and do you get this information?

c. What are you good at in terms of patient safety?

d. What do you experience the healthcare professionals emphasize in terms of quality and patient safety – and what are your thoughts on their perspectives?

e. What do you experience the patients and their families emphasize in terms of quality and patient safety – and what are your thoughts on their perspectives, and how do you adapt the healthcare services accordingly?

f. Can you give examples of how homecare adapt care and services?

g. Do you focus on innovation in your work, and if yes, how?

## 4. Sensemaking and leadership

a. What do you emphasize when you speak of risks, patient safety risk and adverse events?

b. Is there a difference in how leaders and employees talk about risk, and can you elaborate on this with some examples?

c. Is there a difference in the concerns leaders and healthcare professionals have regarding quality and patient safety, and can you give examples of this?

d. Is there a difference in the way you talk about risk with leaders/managers higher up in the system than with those who perform the healthcare services in practice, and can you give examples of how you adapt or experience the difference?

e. Do you use the term risk, or do you prefer other terms? What other terms do you use that include risk?

## 5. Social amplification of risk

a. What do you experience that the authorities emphasize in terms of quality and patient safety - how do you experience this perspective?

b. What do you experience the media emphasizes in terms of quality and patient safety - how do you experience this perspective?

c. What are the healthcare professionals’ concerns about the quality of healthcare provided, and how do you respond to this?

d. Are there any risks that get more attention than others, and why is that?

e. Do you worry that there are risks that you oversee, and what do you do to make sure you don’t?

f. Has the way you talk about the risk of patient harm, patient safety and adverse events changed in any way – if so, how, and why?

## 6. Wrapping it all up

What have I not asked you, but is relevant and important for me to know?
